# Clinical Report on Cases Observed at the New Orleans Charity Hospital

**Published:** 1859-09

**Authors:** Austin Flint

**Affiliations:** Prof. of Clin. Med., &c., in the New Orleans School of Medicine


// NEW YORK MONTHLY REVIEW,

AND

BUFFALO MEDICAL JOURNAL.

VOL. 15. SEPTEMBER, 1859. NO. 4.

### ORIGINAL COMMUNICATIONS.

ART. I.—*Clinical Report on cases observed at the New Orleans Charity Hospital.* By AUSTIN FLINT, M.D., Prof. of Clin. Med., etc., in the New Orleans School of Medicine.

### TYPHOID FEVER.

THE number of cases of Typhoid Fever admitted into my wards during my term of service, extending from the middle of November, 1858, to the middle of February, 1859, was not large. My clinical records contain the notes of only nine cases, which were all that came under my observation. In my report on these cases, I shall follow the plan pursued in my two previous reports, giving an account of each case separately, condensed from the records made daily at the bed-side, and appending a few remarks suggested by a reviewal of the interesting points embraced in the history of the case.

Much discussion has taken place within the last few years respecting typhoid fever, as occurring in the southern and western portions of our country. Doubt and difference of opinion have arisen often, in various sections, as to whether prevailing forms of fever were truly typhoid, or merely varieties of remitting fever. Considerable confusion proceeds from practitioners and writers not always appreciating, or keeping clearly in view, the nosological distinction between these two types of fever. Typhoid fever, as studied by Louis, and its individuality determined by the only method in which the natural history of a disease can be fully ascertained, viz., the analyses of a sufficient number of recorded cases, presents a group of characters which has been found to be identical, when the disease has been studied after the same method in different seasons and countries. It is a striking and significant fact, that the typhoid fever of Paris is in all respects the typhoid fever of London, and of certain parts of this country ; the various symptomatic phenomena and effects, in a series of cases recorded and analyzed by different observers, being found to occur in nearly the same arithmetical ratio. The same is true of the disease as occurring in the same place in different years. For the sake of clearness, to this disease the name of *typhoid fever* should be restricted. The term, however, is often used in a more comprehensive sense, to embrace cases of remitting fever in which more or less of the symptoms of typhoid fever, especially those pertaining to the nervous system, are manifested ; in other words, typhoid fever and a typhoid state, terms nosologically quite different, are often considered as synonymous. Typhoid fever is a disease distinguished by a peculiar group of abdominal and other disorders, a characteristic eruption in a large proportion of cases, certain laws relating to sex, age, season, duration, etc., in addition to those phenomena relating to the mind and nervous system with which the name typhoid is commonly associated. Remitting and other forms of fever may present the same symptoms, that is, a typhoid state, while they are sufficiently distinguished by other characters from typhoid fever. This distinction should be clearly apprehended, and is important to be borne in mind in talking or writing on the prevalence, etc., of typhoid fever.

Confusion in the application of the term typhoid fever, and difference of opinion among practitioners as to whether certain cases of fever be typhoid or not, belong especially to those parts of the country in which periodical fevers prevail to a greater or less extent. There is a reason for this, in the fact that it is sometimes difficult, clinically, to distinguish between typhoid and remitting fever. There is an excellent reason for this difficulty, viz., typhoid fever and periodical fever may be blended. The most rational view of remitting fever, as it seems to me, is to regard it as an affection generated by the combined germs of these two forms of fever. This view reconciles and explains facts which, otherwise, cannot be satisfactorily accounted for. It is intelligible that practitioners should hold pertinaciously to the doctrine of remitting fever running into typhoid ; for, in certain cases, so many of the phenomena of the latter are presented, as to render the differential diagnosis doubtful or impossible. The great diversity in the symptomatic history of different cases of remitting fever, can be understood when it is considered that the morbid effects of the typhoid element may be mingled with those of the periodical element in every variety of proportion. That typical cases of typhoid fever are rarely seen in malarious districts, is simply because the disease is more or less modified by the influence of malaria. That typhoid fever replaces remitting fever in proportion as malarious emanations from the soil are extinguished by cultivation, and, finally, becomes the indigenous form of fever in sections where it had been previously unknown, is consistent with this doctrine of the blending of periodical and continued fever. Is it probable that the special cause of typhoid fever remains in abeyance precisely so long as periodical fevers prevail, and manifests itself directly the latter cease to be prevalent? Is it not a more probable supposition that the special causes of the two fevers have co-existed, and that the characters of typhoid fever have been, to a greater or less extent, masked by the combination.

But I have pursued this train of remark farther than I had intended. With reference to the blending of fevers, as well as other questions, the fevers of those sections of the country in which malaria is more or less abundant, afford scope for greater clinical study than has as yet been bestowed on them, especially by means of the analysis of recorded cases. It is not claimed for this report that it contributes much toward the natural history of typhoid fever as presented at the South. The cases are too few for numerical analysis. Apart, however, from interesting points which belong to the histories of the cases individually, they will at least show that well-marked examples of this form of fever, not differing in essential characters from cases in northern cities, are sufficiently common in New Orleans.

Case 1. *Non-suppurating parotitis during convalescence from typhoid fever.*

John Hughes, aged 25, admitted Nov. 1, 1858.

This patient had been fifteen days in hospital when my service commenced. Convalescence was dated from Nov. 21. After the case came under my observation, there existed active delirium and dejections in bed. The treatment consisted of milk-punch and essence of beef, prior to convalescence; and, during convalescence, the citrate of iron and quinine. The latter remedy was prescribed in consequence of the anæmic appearance of the patient. He had been subject to attacks of intermitting fever; but the last attack was six months prior to his present disease.

Nov. 29th, eight days from the date of convalescence, swelling of the right parotid commenced. It became considerably swelled, painful and tender on pressure. There was no febrile movement, and the patient, continued to sit up. The swelling was painted with the tincture of iodine, and the water-dressing applied, the citrate of iron and quinine being continued. The swelling, redness, and tenderness persisted for several days, then gradually diminished, and had disappeared on Dec. 1st., when he was discharged, complaining only of weakness.

*Remarks.*—The history of this case was incomplete, in consequence of its having been under my observation only during the latter part of the febrile career. The only point of interest is the occurrence of parotitis during convalescence.

Parotitis occurs in a certain proportion of cases, in connection with continued fever. It is rarely referred to by writers, and when mentioned has been considered as an event incident to convalescence. It has been included among the events called critical. It has, however, no claims to this character; and, so far as my observations go, its occurrence during convalescence is an exception to the general rule. Of six instances embraced in my reports of the analysis of 164 cases of continued fever, published in 1852, in four the parotitis became developed during the progress of the fever. The instance now reported, offered an exception to the rule. So far from tending to bring the fever to a favorable termination, it constitutes a serious complication, not only adding to the sufferings of the patient, but increasing the severity and danger of the disease.

Of the six cases just referred to, in all save one the inflammation proceeded to suppuration; and in the excepted case, death occurred before sufficient time had elapsed for suppuration to take place. I do not recollect to have met with an instance, aside from that now reported, of parotitis with considerable swelling, pain, and redness, developed in connection with continued fever, and disappearing by resolution. Is it fair to attribute this rare mode of termination in the present instance, to the remedial measures employed? These were simple, viz., the application of the tincture of iodine for one or two days, followed by the water-dressing, the patient continuing to take the citrate of iron and quinine, with full diet. Without denying to them a certain amount of influence, it must, I think, be admitted that it is more rational to consider the non-suppuration as due to the intrinsic tendency of the local affection in this instance, rather than to the efficiency of the therapeutical means employed.

What relation does parotitis, occurring as a complication or sequel of typhoid fever, sustain to the fever? It is an event of too rare occurrence to be considered as being closely connected with the disease, but it occurs sufficiently often to denote something more than coincidence. It is a contingent, and yet not purely an accidental event. My analytical researches show that there is a proneness to its occurrence at certain periods, and not at other periods. It occurred in five of thirty cases of continued fever recorded between March 29, 1849, and May, 1850. Prior to this period, an instance had never fallen under my observation. From this period to April, 1852, I recorded one hundred and twelve cases, which I afterwards analyzed; and out of this number parotitis was developed in but a single instance. I do not remember that among the cases which I have recorded during the last six years, numbering probably one hundred, or more, an instance has occurred excepting the case now reported. Concerning the nature of its relation to typhoid fever, it would be useless, with our present knowledge, to inquire.

CASE 2. *Typhoid fever complicated with subacute, latent pneumonia.*

Peter Boyle, aged 25, admitted Nov. 17, 1858.

He had kept the bed two days before his admission, and had not been well for the previous week. The symptoms of the access were, headache, nausea and vomiting, occasional looseness of the bowels, sense of fatigue, etc.

After his admission, he presented symptoms characteristic of typhoid fever, as follows:—slowness of the intellect, and apparent indifference, but no manifestations of delirium. He replied to questions coherently, but was sometimes unable to tell the day of the week, or the time of the day. The abdomen was moderately tympanitic and tender in the iliac regions. Moderate diarrhœa continued. Sordes appeared on the teeth. A small number of rose spots were observed over the chest and abdomen, oval, papular, and the redness momentarily disappearing on pressure. On the sixth day after admission, there were, dullness on percussion over the lower portion of the chest on the right side, the upper boundary corresponding with the interlobar fissure, and relatively feeble respiration. The dullness on the following day became marked, and the respiration continued feeble, without development of the bronchial characters. The cough and expectoration were slight; it is not mentioned that the sputa were rusty; the pulse and respiration at this time were not accelerated, and convalescence was declared thirteen days after admission. The pulse did not rise at any time over 90, and about the time of convalescence fell to 68. He was discharged, complaining only of slight cough, nine days after the date of convalescence, having been twenty-two days in hospital.

The treatment consisted of small doses of the sulphate of morphia, from time to time, to relieve the diarrhœa; brandy, in doses of half an ounce, three times daily at first, afterwards increased to an ounce every two hours; and for diet, the essence of beef and milk.

*Remarks.*—As regards the events belonging to the clinical history of typhoid fever, this case presented no unusual features. It was a case of less than medium severity as regards ataxic or nervous symptoms, and much milder than the average as regards disturbance of the circulation. The development of pneumonitis is a point of interest. This is not a very rare complication of typhoid fever. It occurs at different periods of the febrile career, and cases differ remarkably in the symptoms and general disorder attributable to it. When it occurs early in the disease, and its symptomatic phenomena are marked, the practitioner, without due care, is liable to fall into the error of regarding the pneumonia as the primary or sole cause of the affection. A certain proportion of the cases of so-called typhoid pneumonia, are cases of typhoid fever with pneumonic inflammation developed secondarily; the distinction between pneumonia associated with certain typhoid symptoms, and typhoid fever complicated with pneumonia, not being always clearly apprehended. On the other hand, the occurrence of pneumonia in the course of typhoid fever, is sometimes overlooked. It can hardly be otherwise, if the practitioner rely upon symptoms, to the exclusion of physical signs. Pneumonia, as a primitive affection, is sometimes remarkably latent; pain, accelerated breathing, rusty sputa, and even cough, being wanting. This is not unfrequently the case when it is developed in connection with typhoid fever, or some other affection. I do not now refer to the instances of pseudo-pneumonia in which, during protracted fever with adynamia, hypostatic congestion, œdema, and perhaps a low grade of inflammatory action, occur in the latter stage of the disease; but I have reference to distinct pneumonic inflammation extending over a lobe, and giving rise to the solidifying exudation. In these latent cases, the inflammation may be distinguished as subacute; but not chronic, for resolution may take place rapidly. The auscultatory signs are apt to be less marked than in cases of ordinary, frank pneumonia.

How does the co-existence of pneumonia affect the treatment of typhoid fever? As a rule, it furnishes a contra-indication for measures which tend to impair the forces upon which dependence is to be placed for a safe passage through the febrile career. Whatever may be thought of the propriety of general and local blood-letting, mercurialization, antimony, etc., when the pneumonia is primitive, the importance of supporting the powers of the system renders these measures inappropriate when the pneumonia is a complication of typhoid fever. As a rule, the co-existence of pneumonia furnishes more than a contra-indication as regards debilitating measures; it constitutes a positive indication for additional sustaining treatment. In proportion as the tendency to death by asthenia is increased by this complication, the safety of the patient depends on the efficient use of alcoholic stimulants and nutriment.

CASE 3. *Typhoid fever complicated with pneumonitis. Sloughing over hips and sacrum. Probable enlargement of bronchial glands. Tedious convalescence.*

Henry Corcoran, aged 28, Irish, laborer, admitted Nov. 24th, 1858.

He stated that he had had a severe cold for four weeks, but had kept about until four days before his admission, when he took to the bed.

The symptoms and signs of pneumonia affecting the lower lobe of the left lung, were present on his admission. The respirations were 32. There was circumscribed redness of the cheeks. The expectoration was adhesive and rusty. Marked dullness on percussion existed over the lower lobe of the right lung, with crepitant râle and the bronchial whisper.

Delirium soon became a prominent symptom, consisting of incoherent talking, and frequent efforts to get out of bed, in which, when unobserved, he sometimes succeeded, and on one occasion put on his clothes. The respirations were increased to 48. The pulse rose to 100. There was moderate diarrhœa, together with tympanites and tenderness in the iliac regions; the teeth were loaded with sordes. The tongue became dry and hard. In the progress of the febrile career, coma-vigil, carphologia, and subsultus were developed. An eruption in this case was not observed.

Improvement was apparent Dec. 2d, eight days after admission, and twelve days after taking to the bed. The prostration, however, was extreme. Sloughing occurred over the hips and sacrum. The patient presented a cadaveric appearance. He was distinctly convalescent in three or four days afterward, but the convalescence was extremely tedious. The ulcerations over the hips and sacrum healed very slowly, and he was greatly annoyed by a dry, teazing, spasmodic cough. He remained in hospital to the end of my service, and afterward. The cough had ceased, the sores had healed, and he was gaining steadily in strength and weight.

Examinations of the chest during convalescence showed complete resolution of the pneumonia; the dullness on percussion over the affected lobe having disappeared, and the normal respiration returned. But there was marked dullness over the inner half of the infra-clavicular region of the right side, the respiratory murmur being, in this situation, relatively feeble, without the bronchial characters. These signs at first led me to suspect tuberculosis; but, in view of the absence of bronchial or broncho-vesicular respiration, and the progressive improvement of the patient, enlargement of the bronchial glands seemed to be a more rational supposition.

The treatment during the career of the fever consisted of small doses of the sulphate of morphia, when the dejections were frequent; brandy in milk-punch, which was given in doses of an ounce (*i. e.* of brandy)

hourly; and essence of beef at short intervals. During convalescence, opium and the sulphate of morphia were prescribed freely, to relieve the cough, and the chlorate of potassa was given, in doses of a scruple three times daily.

*Remarks.*—In this case, pneumonia existed when the patient came under observation, four days after taking to the bed. The symptoms, as well as signs, were sufficiently marked. The patient was not in a condition to give an intelligible account of the previous history. In such a case, if the practitioner be satisfied when he has discovered a disease which will account for many of the morbid phenomena, he is liable to go no further in his diagnosis than the pneumonia. And in this case, the eruption of typhoid fever was wanting. Yet, exclusive of the eruption, the symptoms characteristic of typhoid fever were present. On the other hand, the general symptoms were out of proportion to the local affection, and persisted while the resolution of the pneumonia was going on. The diagnosis of typhoid fever complicated with pneumonia, was positive.

The case was one of severity and danger. For several days a fatal termination was confidently predicted by some of those who were accustomed to visit the wards. I cannot doubt that the life of the patient was preserved by the efficient employment of brandy, with concentrated nourishment. The importance of these measures in the treatment of typhoid fever, is no novelty in English and American medical practice, but it is not yet fully appreciated by the majority of practitioners. That the pulse falls and is prevented from rising in frequency, in some cases, by the administration of from one to two ounces of brandy hourly; and that the free use of spirits is not only consistent with, but may be rendered still more important by, pneumonia or some other inflammatory complication, is an innovation in therapeutics so much at variance with traditionary opinions, as not to be readily adopted without the evidence of personal observation. Experience, however, furnishes abundant proof that, in comparison with sustaining measures in the treatment of typhoid fever, other known remedial agencies are of small importance.

The probable enlargement of the bronchial glands, as denoted by the physical signs taken in connection with the symptoms, is an interesting point in the history of this case.

CASE 4. *Typhoid fever with predominance and persistence of the abdominal symptoms.*

Frank Schuneman, aged 23, German, laborer, resided in New Orleans only six weeks; admitted Nov. 27th, 1858.

He stated that he had not been well for a week before his admission, complaining of pain in the head, lassitude, anorexia, diarrhœa, epistaxis, etc.; but he did not take to the bed prior to his admission.

He presented on his admission, frequent dejections, marked tenderness and gurgling in the iliac regions, and moderate febrile movement.

The abdominal symptoms were prominent during the febrile career; the dejections were frequent; tympanites and tenderness marked. These symptoms continued into convalescence. Delirium was not manifested. The febrile movement was not great. Epistaxis occurred frequently, taking place for several successive days. An eruption of rose spots was well marked.

The patient could not be pronounced convalescent till Dec. 20th, twenty-four days from the day of admission and the time of taking to the bed; but prior to this date, he was for several days apparently on the verge of convalescence, on some days even sitting up a little. Convalescence appeared to be postponed by the persistence of the abdominal symptoms.

He was discharged Dec. 28th, after being in hospital thirty-two days, feeling well enough to take a journey north to his friends.

The treatment consisted of opium by the mouth and by enema, and the moderate use of brandy in milk-punch, with essence of beef.

*Remarks.*—The details of this, as well as the other cases, were fully recorded at the bed side; but it would be tedious and useless to reproduce them in this report. My object is only to give a sketch of the history sufficiently to show the correctness of the diagnosis, and to present any particular points of interest.

This case illustrates a class of cases of typhoid fever, distinguished by the prominence and persistence of the characteristic abdominal symptoms. In all other respects the phenomena of the disease may denote mildness. The febrile movement may be moderate, ataxic symptoms wanting, the prostration not great; but the dejections are frequent, the abdomen distended, and tenderness in the iliac regions marked. These predominating symptoms point to an unusual abundance or deposit in the intestinal (Peyerian) glands, and a corresponding amount of sloughing and ulceration; in other words, the symptoms, as a rule, represent the degree or extent of the lesions within the small intestine, which constitute the anatomical characteristic of typhoid fever. These lesions, when excessive, add considerably to the severity and danger of the disease. They postpone and prolong the period of convalescence. They may prove fatal, even after the patient has passed through the career of the fever, by inducing exhaustion. It is a curious fact that, under these circumstances, the liability to perforation of the intestine is diminished. Statistics show that this rare accident is less likely to occur in severe than in mild cases of the disease.

In a certain proportion of the cases of typhoid fever which end fatally in spite of judicious and efficient management, the result is due to incurable intestinal lesions. There is no special treatment to be employed with a view to prevent their occurrence, or to lessen their amount. Their variation in different cases depends on a determining influence intrinsic to the disease, and beyond our control as much as the eruption in small-pox is more or less abundant without regard to medicinal interference. The indications for treatment embrace only palliative and sustaining measures.

Case 5. *Typhoid fever without any unusual features.*

Alexander Campbell, aged 20, stage driver, admitted Nov. 30, 1858.

He stated that he had been ill about two weeks before his admission, and had kept the bed for a week. His symptoms before admission were, headache, lassitude, chilly sensations at times, and diarrhœa.

On his admission he was somnolent, but easily roused, and replied to questions coherently. There was moderate febrile movement. The abdomen was meteorized, tender in the iliac regions, and he had four or five liquid dejections daily. Numerous rose spots were scattered over the chest and abdomen.

During the febrile career, the several classes of symptoms were marked, but in due proportion; and there were no unusual features. The febrile movement continued to be moderate. The abdominal symptoms were not urgent. Delirium was occasionally manifested by incoherent talking and attempts to get out of bed.

Convalescence was not distinctly declared until Dec. 17th, eighteen days after admission, and twenty-five days from the date of taking to the bed. He was sufficiently well to be discharged Dec. 29th, having been in hospital thirty days.

The treatment consisted of small doses of opium by the mouth, an occasional enema of laudanum; milk-punch, and the essence of beef.

*Remarks.*—This was a case which exemplified all the more important of the characteristic symptomatic phenomena of this form of fever, the symptoms being evenly balanced, and the career of the fever protracted beyond the average duration. In such cases, sustaining measures of treatment being employed, and graduated by the amount of prostration and their effects, the prognosis is always favorable. The patient is, however, exposed to certain accidents which may determine a fatal result. The more important of these are, apoplectic coma, perforation, and intestinal hæmorrhage. But statistics show that these events occur in only a small proportion of cases, so that the chances of the safety of the patient greatly preponderate. It may be laid down as a practical rule, that if the various classes of symptoms which compose the clinical history of typhoid fever, *i. e.*, the symptoms referable to the abdomen, the nervous system, the circulation, etc., are in a proper relative proportion,—in other words, none being excessive—recovery may be expected with proper management, without reference to the duration of the disease. In the cases in which the disease tends to a fatal result, exclusive of those in which death is owing to the accidents just named, certain classes of symptoms are usually disproportionate to the other classes; for example, the abdominal symptoms may preponderate, as in Case No. 4. In like manner, nervous or ataxic symptoms are out of propoition when cases present active delirium, subsultus, carphologia, etc. These cases are apt to prove fatal. Other cases are distinguished by excessive diminution of the vital powers, or adynamia, as shown by frequency and feebleness of the pulse, muscular prostration, etc. The danger in these cases is great. On the other hand, all the symptoms may be so marked as apparently to invest a case with considerable gravity, without much risk to life, provided the symptoms are in due relation to each other. The duration of the disease appears to be determined generally by its intrinsic limitations, and is independent of the circumstances which tend to a fatal termination.

It should have been mentioned in the sketch of this case, that slight sloughing occurred over the hips. This is an accident liable to occur in cases in which the disease is protracted; and it may often be prevented by proper attention and care.

Case 6. *Typhoid fever with intermittent delirium, and tardy convalescence from mental apathy.*

Fred Flukeyer, Swiss, aged 31, stone-mason, admitted Dec. 4th, 1858.

He had not been well for two weeks before his admission, but did not take to the bed till he entered the hospital. The symptoms during the access were, pains in the limbs and abdomen, lassitude, diarrhœa, &c.

On his admission he had moderate febrile movement, tympanitic abdomen, tenderness and gurgling, and diarrhœa. Numerous characteristic rose spots were scattered over the abdomen and chest.

During the progress of the disease, delirium occurred at irregular intervals of two or three days, the patient, during the night, talking incoherently and attempting to get out of bed, and at other times remaining quiet and apparently rational. The febrile movement was not excessive; the abdominal symptoms were moderate, and the career of the fever was not marked by any unusual features. The patient, however, remained, day after day, apparently about to convalesce, but convalescence not distinctly declared. He showed small desire to take food, and no disposition to make any exertion, lying in an apathetic condition for several days after febrile movement and other symptoms of the disease had disappeared. Under these circumstances, he was taken out of bed and made to sit up. From this time the convalescence was distinct and rapid. He was discharged quite well, Jan. 10th, 1859, having been in hospital thirty-six days.

The treatment in this case during the progress of the fever, consisted of small doses of morphia, milk-punch, and essence of beef. During convalescence, the chlorate of potassa was prescribed, in doses of a scruple three times daily.

*Remarks.*—An interesting point in the history of this case is referred to in the following note appended to the record : "This case illustrates the importance, in certain instances, of rousing patients to voluntary exertion. The patient lay day after day in bed, never speaking, and scarcely moving. He seemed almost inanimate, manifested no desire for food, and occasionally vomited. I directed the nurse to take him out of bed, and cause him to sit up for a little while daily. The first time, he fainted. He was near fainting the second time, while I was in the ward. However, the plan was persevered in, and frictions made over the body. In a few days he manifested improvement, and has since convalesced rapidly."

The intermittent delirium is also an interesting point in the history of this case.

Case 7. *Typhoid fever succeeding intermittent fever. Adynamia. Weakness of the first sound of the heart. Persisting frequency of the pulse in convalescence.*

John Meehan, aged 24, admitted into the ward Jan. 9th, 1859.

This patient was subject to relapses of intermittent fever, and had recently been in the ward for an attack of that disease. After being discharged, he was retained in the hospital as an out-door laborer. He came to the ward a few days before his admission, to obtain medicine for a relapse of intermittent fever of the tertian type. The paroxysms were suspended by the sulphate of quinia; but he continued ill, and when no longer able to keep about, he entered the ward and took to the bed.

On his admission, the pulse was 100, and respirations 24. He presented deep capillary congestion of the face, especially of the cheeks, differing from the circumscribed flush of pneumonia in the gradual shading off of the redness. The mind was remarkably slow, but he replied to questions coherently.

A well-marked rose eruption was already developed over the chest and abdomen. The abdomen was meteorized, tender in the iliac regions, and he had moderate diarrhœa.

During the career of the fever, delirium became developed, but in a moderate degree, manifested by incoherency and occasional efforts to get up. The muscular prostration was marked. It was difficult for the patient to protrude the tongue or to speak intelligibly. Twitching of the muscles of the face was observed, but no subsultus at the wrists. The abdominal symptoms continued to be moderate. The pulse varied from 100 to 120, and was notably soft and feeble. The first sound over the apex of the heart was extremely feeble and valvular, the second sound being accentuated in this situation. As the case approached toward convalescence, the first sound became stronger, and finally accentuated at the apex. The patient could not be considered as distinctly convalescent until January 30th, twenty-one days after the date of admission; but for several days the mind had been clear, and all the symptoms denoted the termination of the febrile career, except that the pulse remained frequent. On the 27th of January the pulse was 120; it was 88 on the 30th, when convalescence was distinct. Four days afterward the patient was able to sit up. Convalescence went on without any untoward symptoms, and the patient was discharged quite well February 23, having been in hospital forty-four days.

The treatment in this case consisted of the sulphate of quinia, gr. v. with opium, gr. i., three times daily, for several days; opium by the mouth, and enemas of laudanum from time to time; brandy quite freely, two ounces hourly being given for several successive days; and beef-essence and chicken broth for diet.

*Remarks.*—In this case typhoid fever occurred in a person subject to intermittent fever, and followed on the heels of an attack of the latter affection. I have occasionally met with similar instances of the close succession of the two forms of fever. The intermittent fever was promptly arrested by the special remedy for that disease. Had this remedy not been employed, it is highly probable that the paroxysms would have continued, masking the development of the typhoid fever, and presenting an example of remitting fever apparently running into typhoid fever. It is probable that the blending of intermittent and typhoid fever serves to mask, to a greater or less extent, the phenomena distinctive of each affection. But this, and other interesting points of inquiry, as well as the question whether remitting fever consists of intermittent or typhoid blended, are to be settled by future clinical researches.

This case was distinguished by the preponderance of symptoms denoting adynamia. The patient was greatly prostrated, and the free use of an alcoholic stimulant seemed to be necessary in order to obviate the tendency to death by asthenia. For several days, two ounces of brandy were given hourly, or forty-eight ounces during the twenty-four hours. I have included in the sketch of the history of this case the fact, that the first sound of the heart was weakened. Dr. Stokes, some years since, called attention to the value of this sign as expressing the state of the vital forces, and furnishing an indication and guide for the use of wine or spirits. Dr. Huss, of Stockholm, has also written on the subject, in support of the views of Dr. Stokes.

I can bear testimony to the usefulness of the auscultation of the heart-sounds as regards this application. The power of the heart's contraction may be considered as representing, in typhoid fever, the state of the vital forces; and the relative intensity of the first sound at the apex, is a good criterion of the power with which the heart contracts. In health, the first sound at the apex is relatively more intense than the second; in other words, it is strongly accentuated. It is compound, being made up of the sound produced by expansion of the auricular valves, and of a sound caused by the impulsion of the apex against the walls of the chest. The latter which I have termed the *element of impulsion*, depends, other things being equal, on the power with which the left ventricle contracts; hence, this element is weakened, and may be extinguished when the muscular action of the organ is enfeebled. The valvular element of the first sound is normally less intense than the second sound; so that in proportion as the element of impulsion is impaired, the first sound becomes weaker than the second, and at the same time shorter, and valvular in quality like the second sound. This modification is observed in cases of typhoid fever characterized by adynamia. Auscultation should be practiced over the apex, and in proportion as the first sound is weak, as compared with the second sound, and short and valvular, it is to be inferred that the vital forces are lowered, and sustaining measures are indicated. This sign is more reliable than the pulse; but the latter furnishes corroborative evidence, in its frequency, softness, and feebleness. It is more available, by far, than the evidence of weakness derived from the voluntary muscles. The condition of the mind in cases of typhoid fever is often such that it is impossible to test the strength of the voluntary muscles; and even if this difficulty does not exist, the power of the acts performed by these muscles depends in a measure on the force of the will, and is not, therefore, a safe criterion of the state of the muscular system. The action of the heart being involuntary, it denotes the vital condition of an organ which, aside from the immediate effects of its condition on the circulation, is a representative of the muscular system and a thermometer of the vital forces; and the test of the vital condition of this organ furnished by the intensity of the first sound, has the advantage of being readily available as well as reliable.

Another point of interest in the history of this case is, the frequency of the pulse at the time of convalescence. This is sometimes observed when all the other symptoms appear to denote that the career of the fever is ended. On the other hand, it is oftener the case that at this period the pulse falls below the healthy standard, and rises to its normal frequency as convalescence is established. The condition of the pulse is highly important in watching the progress of typhoid fever; but, isolated from other symptoms, it would sometimes mislead.

CASE 8. *Apoplectic coma developed during the career of typhoid fever. Fatal.*

Warren C. Stovall, aged 19, mail-driver, admitted January 21st, 1859.

As the notes of this case do not occupy much space, I shall transcribe them in full.

Jan. 22. The patient states that he has been confined to the bed five days, and was not well for at least five days before taking to the bed. His symptoms have been diarrhœa, some vomiting, pain in the head, chills, lassitude, anorexia. No epistaxis. Owing to his prostration and mental condition, minute details pertaining to the previous history are not obtained.

The general aspect of the patient is characteristic:—he is somnolent, but easily roused; he has an air of confusion when questioned, and replies slowly after considerable hesitation. During the night he was delirious, attempting often to get out of bed. On one occasion he gave as a reason for getting up, that he was following some black squirrels. He has had no dejection since his admission last evening. The tongue is protruded imperfectly, after a strong effort. It is dry and covered with scabby incrustations. Sordes on the teeth. Abdomen not distended. Moderate tenderness in both iliac regions, and none over the epigastrium. No eruption. The pulse is 128, quick, vibratory. The first sound of the heart over the apex is intense, and the element of impulsion is marked. Respirations are 36. *The inspirations are slightly spasmodic. The attention of the class at the bed side, was called to this symptom and its significance.* Clear percussion-sound over the chest. Slight capillary congestion of face.

Treatment: opium, gr. i, every four hours. Essence of beef and milk for diet.

Jan. 23. The patient is unconscious, and cannot be roused. The countenance is Hippocratic. The inspirations are spasmodic; the respirarations are 29. The extremities are cold. The pulse is nearly extinct. He was delirious during the night, often attempting to get out of bed.

Death occurred an hour after the foregoing was written, while I was in the ward. The body being removed by friends, an autopsy was not made.

*Remarks.*—Sudden coma, generally proving fatal, occurs in a certain proportion of cases of typhoid fever. It occurred in 9 of 164 cases which I have formerly analyzed.* It is developed without being foreshadowed by any symptoms referable to the nervous system. Somnolency, coma-vigil, &c., do not necessarily precede it. So far as our knowledge of it is concerned, it is an accidental event; and it is as likely to occur in mild as in severe cases. It may happen at any period during the febrile career. The pathological condition of the brain on which it depends, is not ascertained. It does not involve inflammation, nor extravasation, nor a notable degree of congestion. To say that it arises from a dynamic change, is saying simply that the condition is not within the scope of morbid anatomy, or, at least has not as yet been revealed by the scalpel. The liability to this accident is always to be taken into account in the prognosis of typhoid fever.

The occurrence of sudden coma is frequently preceded for several hours by an alteration in the rhythm of the respiration. The inspiration is shortened and quickened, or, in other words, spasmodic. This symptom may be present when there are no other symptoms pointing to this accident, or to immediate danger. This altered rhythm of respiration, if not connected with any pulmonary complication, is always to be regarded as an ominous symptom in the course of typhoid fever. If it be present in a marked degree, and pneumonia, or other pulmonary complications, be excluded by the absence of physical signs, the occurrence of coma may be predicted with great confidence. The case just reported furnishes an illustration of this fact. The probability of coma was the subject of clinical remarks at the bedside. Similar illustrations have repeatedly fallen under my observation, and several cases are detailed in my reports on Continued Fever. The symptom evidently arises from a pathological condition affecting the portion of the encephalon which presides over the respiratory acts, before extending so as to suspend the consciousness. Death takes place usually in these cases from an arrest of respiration, or, in other words, by apnœa; but if there be much prostration, or adynamia, as in this case, the mode of dying is by apnœa and asthenia combined.

Rapid vesication over the nucha, sinapisms to the extremities, and the free use of stimulants, have seemed in some instances to prevent a fatal result.

---

* Clinical Reports on Continued Fever, &c. 1852.

CASE 9. *Typhoid fever, the disease being unusually mild.*

Léon Frechou, Frenchman, aged 29, gardener, admitted January 27, 1859.

He had resided in New Orleans but two months. About two months prior to his admission he had an attack of intermittent fever (tertian type), which was arrested by the sulphate of quinia. He had kept the bed for three days before coming to the hospital.

He presented, on his admission, febrile movement, mild diarrhœa, meteorism of abdomen, tenderness in the iliac regions, and gurgling.

The symptoms during the career of the fever, denoted unusual mildness of the disease. The pulse was pretty frequent, varying from 95 to 110. The abdominal symptoms were moderate. There were no manifestations of delirium; he seemed at times dull, and had an air of indifference, but always replied to questions quickly, and apparently with correctness. The tongue remained perfectly clean during the progress of the disease. The rose eruption over the abdomen and chest was copious, and fresh papules continued to appear up to the time of convalescence.

Convalescence was declared February 13, seventeen days after his admission, and twenty days after taking to the bed. The pulse was 90 at the time of convalescence, all the other symptoms indicating that the career of the disease was ended. The convalescence was rapid.

The treatment in this case consisted of brandy in moderate doses, an occasional laudanum enema, and, for diet, boiled milk and the essence of beef.

*Remarks.*—There were no points in the history of this case, of particular interest. The case furnishes a good illustration of mild typhoid fever, the diagnostic phenomena being well marked.

### *General Remarks.*

The nine cases of typhoid fever which have been reported, occurred in my wards during a service of three months. My wards contained about thirty beds. The means of determining the number of cases occurring during the same period in the other wards of the hospital, are not at hand; but assuming an equal number to have occurred in the service of all the visiting physicians, the whole number of cases would exceed one hundred. The yearly report of the officers of the hospital to January, 1859, showed one hundred and twenty-nine cases of typhoid fever. This form of fever, thus, is sufficiently common in New Orleans. The proportion of cases to the number of patients received, is probably not far from the average in northern hospitals. The disease presents the same symptomatic phenomena as at the North. Judging from the histories of the cases now reported, there are no features peculiar to typhoid fever as observed in New Orleans.

It occurs here, as at the North, during the autumnal months. My service commenced in the middle of November, and of the nine reported cases five occurred during the latter half of this month; one case was admitted December 4th, and the remaining three cases in January. It is probable that of the 129 cases which were received during the year, the greater portion occurred during my term of service. It may be worthy of note that my service commenced just at the close of a yellow-fever epidemic which had been unusually severe and protracted, two thousand seven hundred and twenty-seven cases having been treated in the Charity Hospital. The fatality from typhoid fever, judging from these few cases, is not greater than in northern cities. Of the nine cases, but one proved fatal, and in the only fatal case, death was owing to an event which occurs in a certain proportion of cases in all places, viz., apoplectic coma.

The indications for treatment are the same at New Orleans as elsewhere. The treatment pursued in the cases reported was simple and uniform, but certainly not devoid of efficiency. In all the cases save one* it consisted, in addition to opium or the sulphate of morphia administered by the mouth or rectum in doses sufficient to keep the diarrhœa in check, of sustaining measures, in other words, of brandy and concentrated nourishment. These measures were pressed in proportion to the evidence afforded by the symptoms of the extent to which the vital powers were compromised by the disease. Brandy given, as in one of the cases, in doses of two ounces hourly, becomes a remedy of considerable potency. Given in much smaller quantity it can hardly be considered inefficient by those (still not a few) who, regarding it as an incendiary remedy, withhold it entirely in the management of fevers and inflammations. With our present knowledge of the natural history and pathology of typhoid fever, together with the fruits of therapeutical experience, the indications for efficient treatment are fulfilled by sustaining measures graduated in each case by the state of the vital forces and the tendency to a fatal result by asthenia; and the sustaining measures which meet the indications most efficiently, consist of alcoholic stimulants in the form of wine or spirits, with certain forms of nourishment embracing in concentrated bulk the important alimentary principles. The animal essences and milk furnish these principles best combined for nutrition. The sustaining plan of treatment carried out promptly, judiciously, and, if need be, boldly, together with palliative remedies suited to the circumstances in individual cases, will, I am satisfied, save not a few patients who would otherwise perish from typhoid fever; and when not essential to the safety of life, it conduces to a rapid and complete recovery.

---

* In the excepted case the sulphate of quinia was given at the commencement, the patient having quite recently had an attack of intermittent fever.

